# Differential Leucocyte Counts Which May Assist Diagnosis

**Published:** 1907-07-27

**Authors:** 


### Points in Diagnosis.

### DIFFERENTIAL LEUCOCYTE COUNTS WHICH MAY ASSIST DIAGNOSIS.

There are not a great many diseases in which the differential leucocyte count by itself assists the diagnosis, as in the case of eosinophilia; but there are one or two conditions in which a differential leucocyte count is either indispensable to the diagnosis or else may very materially assist it. Such conditions are:—

| | |
|---|---|
| 1. Spleno-medullary leuchæmia<br>2. Lymphatic leuchæmia | Differential leucocyte count essential, in addition to total leucocyte count |
| 3. Malaria<br>4. Typhoid fever<br>5. Suppuration | Differential count along with total count, sometimes extremely useful |

### Leuchæmia or Leucocythæmia.

The most striking feature of the blood in leuchæmia (or leucocythæmia, as it is also called), is the presence of enormous numbers of leucocytes per cubic millimetre of blood; but there are two main varieties of the disease—namely, (*a*) the *spleno-medullary* form; and (*b*) the *lymphatic* form. It is important to distinguish the two, because the prognosis is very different. Both forms are fatal, but whereas the rule is for a patient with spleno-medullary leuchæmia to live upwards of three years from the time that symptoms lead to diagnosis of the condition, a patient with lymphatic leuchæmia does not as a rule survive as many months as the other does years.

On examining blood films from a case of spleno-medullary leuchæmia, one sees at once that there are large numbers of big mono-nuclear cells which at first sight resemble large lymphocytes. These are the *myelocytes,* which differ from large lymphocytes in that their nucleus is not kidney-shaped but round, and in that their protoplasm is not clear and transparent, but full of dark streaks and granules. There are two varieties of these granules, according as they stain blue or red with such a stain as Jenner's; both varieties of the cell have a single large round nucleus, and the blue-granuled variety, or *ordinary myelocyte,* is much commoner than the red-granuled or *eosinophile myelocyte.* No particular clinical significance attaches to the relative proportions of each, and it is usual, for clinical purposes, to enumerate them both simply as myelocytes. Another abnormal variety of leucocyte is also seen—namely, the *basophile cell,* or *mast cell;* it is only important in that basophile cells are practically absent from normal blood, so that their presence in a film is evidence that there is certainly something wrong with the patient; beyond this they have no clinical significance, though it is important to recognise them. A basophile cell is comparatively small, about the size of a small lymphocyte; the basophile granules are large, but not numerous; and if one sees a cell that looks like a small lymphocyte, except that it contains about half a dozen or more big deep purple-blue granules in the protoplasm around the nucleus, it is a basophile cell. All the normal blood cells are present in the blood of spleno-medullary leuchæmia, but owing to the presence of myelocytes and mast cells the relative proportions of the normal cells in each 100 leucocytes counted must necessarily be different to that in health. The following figures give a comparison between the differential leucocyte counts in a typical case of spleno-medullary leuchæmia and in a healthy person respectively:—

| | Spleno-medullary leuchæmia blood % | Healthy blood % |
|---|---|---|
| Small lymphocytes | 10 | 30 |
| Large lymphocytes | 1 | 8 |
| Polymorphonuclear cells | 45 | 60 |
| Coarsely granular eosinophile cells | 2 | 2 |
| Myelocytes | 40 | absent |
| Basophile cells | 2 | absent |

It will be noted in the above count that the coarsely granular eosinophile cells are still 2 per cent., as in health; it is frequently stated that there is eosinophilia in spleno-medullary leuchæmia; this, however, is not our experience. In regard to the myelocytes, their mere presence is not proof of spleno-medullary leuchæmia, for they may be found in small numbers in several other diseases, notably Hodgkin's disease, pernicious anæmia, splenic anæmia, and lymphatic leuchæmia; it is their presence *in large numbers* which is so typical of spleno-medullary leuchæmia.

In lymphatic leuchæmia the differential leucocyte count is very different, but is even more striking still, as the following comparative counts show:—

| | Lymphatic leuchæmia blood | Healthy blood % |
|---|---|---|
| Small lymphocytes | 90% and sometimes even 97% (bracketed with large lymphocytes) | 30 |
| Large lymphocytes | | 8 |
| Polymorphonuclear cells | 9·5% or less | 60 |
| Coarsely granular eosinophile cells | Often absent | 2 |
| Myelocytes | 0·5% | Absent |
| Basophile cells | Absent | Absent |

The enormous number of lymphocytes is very striking. In most cases these are of the ordinary small variety, but sometimes they are a little larger, though not so large as the typical hyaline large lymphocytes of healthy blood. Some observers term them large lymphocytes, however, and this is why the large and the small are bracketed together in the example given above. True large hyaline lymphocytes are scarce in the films, and when large lymphocytes are said to be increased in certain cases of lymphatic leuchæmia the cells would seem to be large specimens of small lymphocytes rather than true large lymphocytes.

The symptoms complained of may be the same in both lymphatic and in spleno-medullary leuchæmia, and they are very variable. The patient may merely be feeling weak, short of breath, and out of sorts; or an abdominal lump may have been detected by him or her; or there may be œdema of the legs, or spontaneous hæmorrhage such as epistaxis, purpura, bleeding gums, hæmaturia, and so on; the first trouble may be the inflammation of a serous membrane, such as pleurisy, or peritonitis with ascites; in the early stages it is not usual for anæmia to be a marked feature of the case, though later it may become extreme. The medical man is led to the diagnosis by examining the patient and finding a big spleen. This viscus is enlarged in both forms of leuchæmia, but much more so in the spleno-medullary than in the lymphatic form; in the latter, in addition to the large spleen, there are commonly enlarged lymphatic glands as well, these glands not infrequently being affected all over the body. The liver is frequently much enlarged in both diseases too, the organ being smooth, uniform, and not hard; there is usually slight pyrexia, a systolic bruit both in the pulmonary area and at the cardiac impulse. Without a blood count it is barely possible to exclude other diseases with similar symptoms and signs; for example, Hodgkin's disease, fungating endocarditis, cirrhosis of the liver, splenic anæmia, lardaceous disease, and malaria. If there is a great increase in the number of the leucocytes, leuchæmia is almost certain. This increase is usually more marked in the spleno-medullary than it is in the lymphatic form, 400,0000 leucocytes per cubic millimetre of blood being than it is in the lymphatic form, 400,000 150,000 are more usual numbers in the latter. Nevertheless there are cases of lymphatic leuchæmia in which over 800,000 leucocytes per cubic millimetre have been found; and in spleno-medullary leuchæmia, especially if under treatment, the numbers may not reach 100,000. The absolute numbers of leucocytes will not, therefore, settle the diagnosis between the two forms, though it may be laid down as a rule that if there is general enlargement of lymphatic glands, together with moderate enlargement of the spleen, and a leucocyte count of 100,000 or more, the diagnosis of lymphatic leuchæmia is certain; whilst if there is no enlargement of lymphatic glands, but an enormous enlargement of the spleen, and a leucocyte count of 300,000 or more, the diagnosis of spleno-medullary leuchæmia is almost certain.

### Indefinite Cases.

In such definite cases as the above no differential leucocyte count will be needed; it is in the less definite cases that it is required, as for example:—

(*a*) When there is general enlargement of the lymphatic glands, moderate enlargement of the spleen, and a leucocyte count of 50,000 or less, an inflammatory lesion may have caused the leucocytosis; if in the differential leucocyte count there be no relative increase of the small lymphocytes, the condition is not lymphatic leuchæmia; if, on the other hand, the small lymphocytes are upwards of 90 per cent., the condition is lymphatic leuchæmia.

(*b*) When the spleen is moderately enlarged, no changes can be detected in the lymphatic glands, but the leucocytes number, say, 150,000 per cubic millimetre. Without a differential count, the diagnosis would seem to be spleno-medullary leuchæmia with a prognosis of three years' life; but it sometimes happens that no enlargement of the lymphatic glands can be detected, and yet the condition is lymphatic and not spleno-medullary leuchæmia. The differential count alone can detect these cases.

(*c*) When the spleen is moderately enlarged, together with the lymphatic glands, and there is no leucocytosis, Hodgkin's disease is very probable in such cases, with a comparatively fair prognosis; it sometimes happens, however, that such a case is really one of lymphatic leuchæmia, either in an early stage or else with the leucocytes greatly reduced by some intercurrent affection. Even when such is the case, however, the differential count continues to show a great preponderance of lymphocytes if the condition be lymphatic leuchæmia; so that it is important to make the differential count in cases of apparent Hodgkin's disease in order to exclude the possibility of latent leuchæmia.

(*d*) Sometimes a *mixed* leuchæmia is found; the symptoms, the size of the spleen, the absence of enlarged lymphatic glands, and the presence of great numbers of leucocytes in the blood may all point to a diagnosis of spleno-medullary leuchæmia; a cursory examination of a stained film may seem to confirm this, owing to many myelocytes being seen; but a careful differential leucocyte count may show that the small lymphocytes may also be very numerous, the importance of this being that the prognosis is proportionately less good.

(*e*) When a case of spleno-medullary leuchæmia has been treated by the application of the *x*-rays to the abdomen, over the splenic area, it not infrequently happens that the number of leucocytes per cubic millimetre becomes reduced almost to normal; for example, the number may diminish from 400,000 per cubic millimetre to, say, 25,000 per cubic millimetre, or even less. The patient may come under observation without any notes as to the previous condition of the blood being obtainable, and the diagnosis may appear doubtful. It is remarkable that, even when the total number of leucocytes has fallen almost to normal in this way, the differential leucocyte count still shows a high proportion of myelocytes, so that an examination of a blood film will at once show the condition to be spleno-medullary leuchæmia, and differentiate it from splenic anæmia, in which there is no such prevalence of myelocytes.

(*f*) Every now and then, when a differential leucocyte count is being made for some other purpose, incipient leuchæmia will be discovered. For example, a man of 60 was suffering from acute and extensive eczema, which he had often suffered from before; except for the eczema, he seemed strong and well. There was not even a suspicion that he had anything more than the eczema the matter with him. He had no enlarged spleen, no enlarged glands, no anæmia, no hæmorrhages. A differential leucocyte count of his blood was made, in a routine way, with a view to seeing if his eczema was associated with eosinophilia. The count was a surprise, for it showed a great preponderance of small lymphocytes, though the total number of leucocytes was not excessive. Lymphatic leuchæmia was suspected, and a bad prognosis given; this was rapidly confirmed, for the patient was dead within three months, the blood condition becoming quite typical, notwithstanding the absence of enlarged lymphatic glands.

